# Recent origin of iron oxidation in extant microbial groups and low clade fidelity of iron metabolisms

**DOI:** 10.1128/aem.01662-24

**Published:** 2025-08-12

**Authors:** Erik Tamre, Gregory Fournier

**Affiliations:** 1 Department of Earth, Atmospheric, and Planetary Sciences, Massachusetts Institute of Technology 2167, Cambridge, Massachusetts, USA; 2 Department of Earth Sciences, Dartmouth College 3728, Hanover, New Hampshire, USA; Washington University in St. Louis, St. Louis, Missouri, USA

**Keywords:** microbial iron oxidation, phylogenetics, Bayesian molecular clock, microbial evolution, planetary microbiology, geomicrobiology, natural history, photoferrotrophy, clade fidelity

## Abstract

Reduced iron was abundant in Earth’s surface environments before their oxygenation, so iron oxidation could have been a common metabolism on the early Earth. Consequently, modern microbial iron oxidation is sometimes seen as a holdover from an earlier biosphere, but the continuity of involved lineages or the metabolic process itself has not been verified. Modern neutrophilic iron oxidizers use cytochrome-porin Cyc2 as the initial electron acceptor in iron oxidation. With the protein as a proxy for the metabolism, we performed a phylogenetic analysis of Cyc2 to understand the evolutionary history of this microbial iron oxidation pathway. In addition to known iron oxidizers, we identified Cyc2 orthologs in gammaproteobacterial endosymbionts of lucinid bivalves. These bivalves have a robust fossil record and rely on seagrass meadows that only appear in the Cretaceous, providing a valuable time calibration in the evolutionary history of Cyc2. Our molecular clock analysis shows that extant sampled Cyc2 diversity has surprisingly recent common ancestry, and iron oxidation metabolisms in Gallionellaceae, Zetaproteobacteria, and photoferrotrophic Chlorobi likely originated in the Neoproterozoic or the Phanerozoic via multiple transfer events. The groups responsible for microbial iron oxidation have thus changed over Earth history, possibly reflecting the instability of niches with sufficient reduced iron. We note that frequent transfer and changing taxonomic distribution may be a general pattern for traits which are selected sporadically across space and time. Based on iron metabolism and other processes, we explore this concept of a trait’s “clade fidelity” (or lack thereof) and establish its evolutionary importance.

**IMPORTANCE:**

Bacteria can oxidize iron to produce energy. As there was plenty of reduced iron available on the early Earth and there is only a little today, it was sometimes thought that bacteria that oxidize iron today are a small remnant of a larger group that used to do it. We studied the evolutionary history of the iron oxidation pathway that modern bacteria use, and we found that they developed that pathway relatively recently: whatever did it in the past is no longer around today. It would probably be hard for any group of organisms to keep doing iron oxidation over billions of years since iron availability is so variable: they are likely to go extinct or lose this ability at some point. We suggest this as a general trend in evolution that traits which are only sporadically useful are commonly lost—and then re-invented or re-distributed—or the trait will go extinct.

## INTRODUCTION

Microbial iron oxidation has been proposed as a common metabolism on the early Earth, given the availability of reduced iron in surface environments before their widespread oxygenation (1). Banded iron formations (BIFs) present a particularly voluminous body of geological evidence for the abundance of reduced iron once dissolved in the ocean, and photoferrotrophy—one form of microbial iron oxidation—is often implicated in the origin of these formations, given the lack of oxidative power available to oxidize large quantities of iron chemotrophically (e.g., see references 2 and 3). As BIFs are a quantitatively important presence in the geological record until the Paleoproterozoic, involvement of photoferrotrophy in their creation would suggest a dominant role for that metabolism in early biological productivity. Consequently, modern microbial iron oxidation is sometimes seen as a holdover from a much earlier biosphere (4), yet any continuity of involved lineages or even the metabolic process itself has not been verified. Here, we study the phylogenetic history of Cyc2, a key enzyme in modern microbial iron oxidation, in order to understand the history and antiquity of this pathway as present in modern organisms.

Cytochrome-porin Cyc2 is used by chemolithotrophic Gallionellaceae and Zetaproteobacteria as well as photoferrotrophic Chlorobi as the initial electron acceptor in their iron oxidation pathway (5): it takes electrons from dissolved iron(II) outside the cell and passes them down an electron transport chain which eventually reaches the terminal oxidase (6). Since known ecologically important neutrophilic iron oxidizers have Cyc2 and it is highly expressed in environments with a high degree of microbial iron oxidation, it has been proposed as a reliable genomic marker for this metabolism (5). This allows us to study the phylogenetic history of this protein as a proxy for the history of microbial iron oxidation as observed in modern neutrophilic taxa.

Some previous studies have already considered the possible role of photoferrotrophic Chlorobi in the context of the inference that photoferrotrophy was an important metabolism in the Earth’s past. For example, Thompson et al. (2) propose that they were important players in biogeochemical cycles in the Precambrian, but without explicitly dating the clade or their iron-oxidizing metabolism. Other efforts have estimated the age of photoferrotrophic Chlorobi via molecular dating, with varying results: Ward and Shih (7) found that this clade postdates 300 Ma, while Magnabosco et al. (8) ran analyses with multiple calibration sets, which all suggest an age of around 1 Ga. Now that Cyc2 has been identified as the initial electron acceptor in photoferrotrophic Chlorobi as well as other, chemotrophic iron oxidizers, we attempt to date the Cyc2 phylogeny directly as a proxy for the age of the underlying microbial iron oxidation process, rather than estimating the age of currently iron-oxidizing groups on the species tree. The Cyc2 phylogenetic history will include the photoferrotrophic Chlorobi and offer an estimate for the age of photoferrotrophy in that clade, which is not necessarily the same as the age of that clade, as the metabolism may not always have been inherited vertically. However, our approach also extends the history of Cyc2-dependent microbial iron oxidation backward to the last common ancestor of modern Cyc2 sequences, potentially further into the past than any extant group performing iron oxidation today.

## MATERIALS AND METHODS

### Cyc2 sequence collection and alignment

The Cyc2 sequence in *Chlorobium phaeoferrooxidans* str. KB01 (protein accession number WP_076792910.1) as identified by reference 9 was used to query the National Center for Biotechnology Information (NCBI) non-redundant protein database (10) for homologous Cyc2 sequences using BLASTp (11).

Two different data sets of Cyc2 orthologs were assembled, differing in the breadth of the sampling strategy. A broad sample includes the first 500 BLAST hits returned by the search, exhaustively covering the sequence space in and around the main clades of neutrophilic iron oxidizers as well as including any clades containing possible calibration points in the broader protein family. This large sequence set showed multiple suspected misalignments on visual inspection, especially within very distantly related sequences (alignment files in the supplemental data linked at https://doi.org/10.5061/dryad.905qfttvf), calling for the cross-validation of results against a smaller data set including only the descendants of the last common ancestor of Cyc2 sequences in the modern groups of neutrophilic iron oxidizers: in particular, chemolithotrophic Gallionellaceae and Zetaproteobacteria as well as photoferrotrophic Chlorobi. That narrow sample is a subset of the broad sample comprising 109 sequences (equivalent to what prior work on Cyc2 in references 5 and 6 has called cluster 1 and already identified as corresponding to neutrophilic iron oxidation), and a visual inspection of the resulting alignment showed no obvious misalignments. The alignments were made using MAFFT 7.245 (12), with the automatic choice of alignment algorithm (“mafft --auto”) selecting L-INS-i.

### Cyc2 phylogenetic tree search

Based on the alignments, maximum-likelihood phylogenetic trees were built using IQTree 1.6.3 (13). The ModelFinder function was used to select the best-fitting model, and LG + R7 + F (LG substitution model with seven free rate categories and equilibrium frequencies estimated from the data) was selected based on the Bayesian Information Criterion score. The support for each bipartition was estimated using ultrafast bootstraps (14) with 1,000 replicates. The relationships between taxa involved in the narrow sampling largely reflected their relationships in the tree based on the broad sampling. Nevertheless, given the potential for misalignments in the broad sample to drive spurious substitution rate inferences, only the best tree obtained for the narrow sampling scheme was used as a basis for molecular clock runs. The tree based on broad sampling was still used as a means of outgroup rooting for the narrow sample.

### 16S species tree construction

To explicitly compare the relationships between Cyc2 sequences to species tree relationships, 16S ribosomal RNA sequences from taxa with Cyc2 were recruited from the SILVA ribosomal RNA database (15). Not all taxa with Cyc2 sequences had a 16S sequence available (especially where Cyc2 sequences came from metagenomic studies). Nevertheless, in most clades of iron-oxidizers, the available 16S sequences covered both sides of the clade’s basal split on the Cyc2 tree, so that a congruent crown group could be recovered (see Fig. S1 for complete taxon sampling and the full 16S tree).

The 16S sequences were aligned using MAFFT 7.245 (12) with the automatic choice of alignment algorithm (“mafft --auto”) selecting L-INS-i. Based on this alignment, a maximum-likelihood phylogenetic tree was built using IQTree 1.6.3 (13) with GTR + G4 + F + I (GTR substitution model with four gamma-distributed rate categories, equilibrium frequencies estimated from the data and the presence of invariant sites). The support for each bipartition was estimated using ultrafast bootstraps (14) with 1,000 replicates.

The tree was rooted according to bacterial species tree work in prior literature (e.g., see references 16 and 17), separating Chlorobi from the rest of the diversity in this tree. The root is also consistent with that inferred from minimum ancestor deviation (MAD) analysis (18). Note that while the best root inferred by MAD narrowly falls on a neighboring branch compared to the one that the root has been placed on, MAD only offers a very weak preference between these options: the ancestor deviation values at MAD’s best root position and at the adjacent node connecting the branches in question are identical to the third decimal point (see Fig. S2).

### Bayesian molecular clock inferences

PhyloBayes 4.1 (19–21) was used for the relaxed molecular clock runs. Each clock includes a uniform root prior constrained by 2.4 Ga as an older bound, reflective of the dominating presence of aerobic groups on both sides of the root, for example, *Gallionella* and Zetaproteobacteria use oxygen as the electron acceptor for the very iron oxidation process mediated by Cyc2 (references 22 and 23, respectively). As a younger bound, we used 75 Ma, reflecting that all known lucinid bivalves host gammaproteobacterial endosymbionts as adults (24), and the symbiotic relationship with the chemoautotrophic bacteria would have therefore been established by the lucinid radiation in the late Cretaceous. Thus, as lucinid endosymbiont sequences of Cyc2 represent a subtree of the Cyc2 phylogeny to be dated, the root necessarily predates their divergence. The extreme breadth of the root prior is intended to reflect that lack of prior information on the age of Cyc2-based iron oxidation: that is the question the clock is intended to answer. Nevertheless, note that we reject a priori an Archean root, given the topology of the phylogenetic tree, as discussed in the Results section.

It is also important to note that the exact younger bound on the root in the late Phanerozoic does not have a significant impact on the outcome, as the uncertainty in the younger bound selection represents only a small fraction of the total interval covered by the intentionally largely uninformative root prior. In fact, the choice of tree process prior, together with the internal node calibration, much more dramatically alters the joint root prior than would any small change in the younger bound on the root prior itself (see Fig. S3). A more extensive discussion of the choice of internal calibrations is given in the Results.

Both the birth-death prior process and a uniform prior on node ages were tested as tree priors, and each relaxed clock model was run with each of the prior processes in PhyloBayes 4.1. We included three clock models: (i) an uncorrelated model with rates on each branch drawn from a gamma distribution (“-ugam”), (ii) an autocorrelated model with rates drawn from a log-normal distribution (“-ln”), and (iii) an autocorrelated model with rates based on the CIR process (“-cir”). Thus, six clocks in total were run under the posterior, and at least two chains were required to converge for each clock before the posterior distribution would be sampled. In accordance with recommendations in the PhyloBayes manual, convergence was assessed by requiring TRACECOMP values for all estimated variables to be <0.3 and the minimum effective size of the sample to be >50.

Each clock was also run under the prior without the inclusion of sequence information, in order to check the parameters of the joint prior: for example, the impact of the internal node calibrations and the tree process prior means that the joint prior on the root age is considerably narrower than the root prior alone. That also allowed us to distinguish the impact of sequence data from the constraints imposed by the prior. The prior-only runs did not differ between clocks using the same tree process prior, but a different clock model: this is expected, since the only function of the clock model is to convert sequence substitutions into time, and sequence data do not impact runs under the prior.

Custom Python scripts were used to read the .datedist files from PhyloBayes, and the ggplot2 package in R was used to visualize the full variability of divergence date estimates in the sample. In the figures as well as the text of the article, we refer to bacterial clades using the nomenclature applicable before the changes implemented in 2021 but subject to continuing contention (25, 26). This is largely motivated by the lingering use of the old nomenclature in the NCBI Taxonomy Database as well as in taxon names for sequences uploaded before the change, which make up the vast majority of the sequences in the trees and need to be reconcilable to the main text. Where applicable, we still mention the recent classification upon the first appearance of the clade in the text to allow for reconciliation with the new nomenclature as well.

## RESULTS

### Phylogenetic tree of Cyc2 and its comparison with the 16S tree

**Fig 1:**
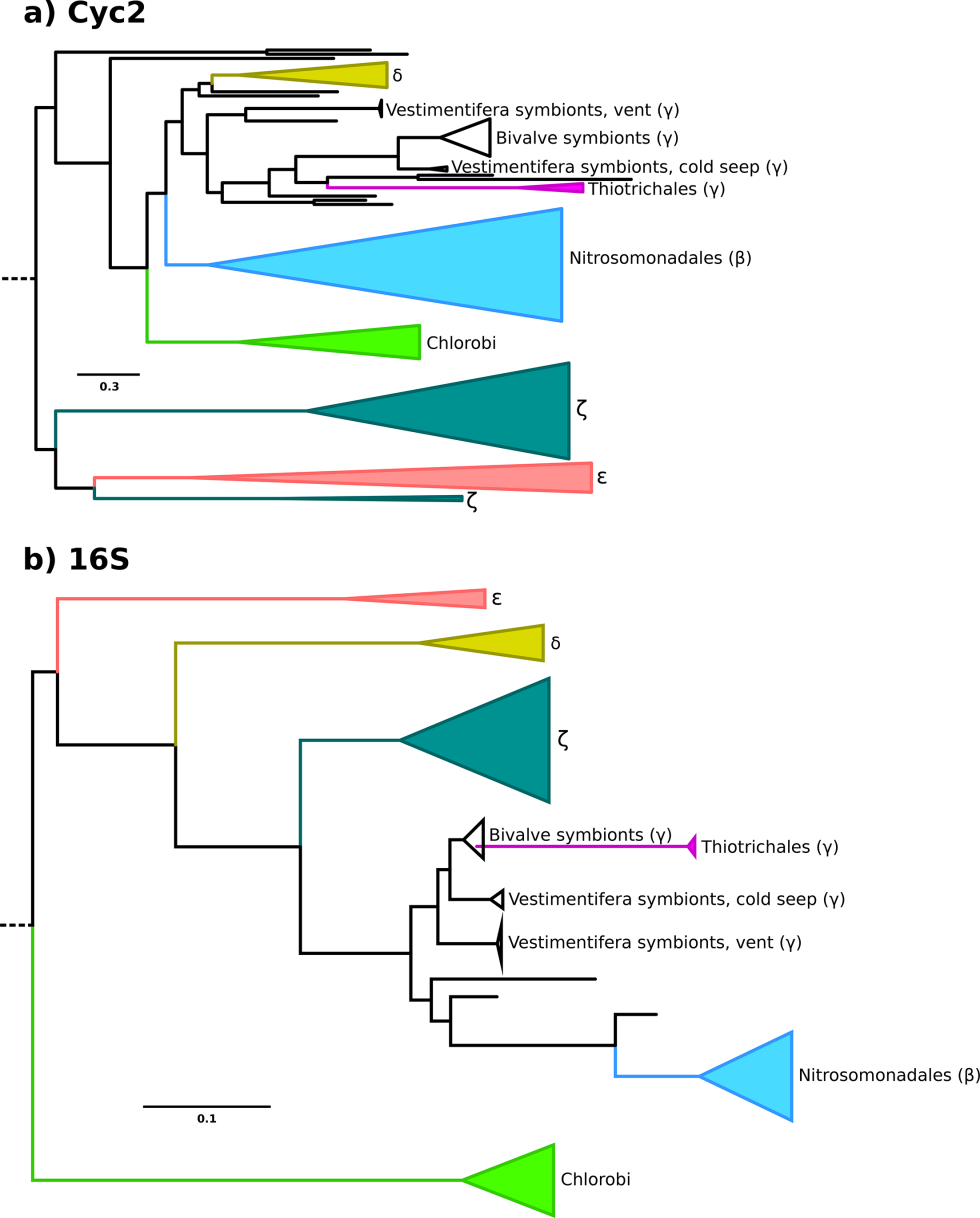
**(a**) Maximum-likelihood tree of Cyc2 proteins, with groups of sequences in closely related organisms collapsed for clarity. (**b**) Maximum-likelihood species tree of taxa with Cyc2 based on the 16S small subunit ribosomal RNA, with groups of sequences in closely related organisms collapsed for clarity. In both panels, Greek letters refer to taxa falling in the following groups: β, Betaproteobacteria; γ, Gammaproteobacteria; ζ, Zetaproteobacteria (all three now classes in phylum Pseudomonadota); δ, Deltaproteobacteria (now phylum Myxococcota); ε, Epsilonproteobacteria (now phylum Campylobacterota). See Fig. S1 and S4, respectively, for the species tree and Cyc2 tree figures with no collapsed nodes, and see supplemental data at https://doi.org/10.5061/dryad.905qfttvf for the annotated tree files.

Figure 1a shows the maximum-likelihood tree of Cyc2 protein sequences, and Fig. 1b shows the species tree of corresponding organisms re-constructed from 16S small subunit ribosomal RNA. The Cyc2 tree does not closely resemble the species tree of corresponding organisms, other than in shallow clades that reflect short periods of vertical inheritance or alternatively transfer between closely related taxa. These shallow clades represent subsets of Chlorobi, Betaproteobacteria (in particular, Gallionellaceae), Zetaproteobacteria, Epsilonproteobacteria, Deltaproteobacteria, and Gammaproteobacteria, in addition to scattered sequences of Muproteobacteria, Lambdaproteobacteria, and Nitrospirae.

Even though there are groups where protein sequences cluster according to the broad affiliation of the organisms, the relationships between these groups on the Cyc2 tree often do not reflect the relationships of organisms on the species tree. For example, the gammaproteobacterial Cyc2 sequences fall sister to deltaproteobacterial sequences with betaproteobacterial sequences as an outgroup, whereas Gammaproteobacteria themselves are much more closely related to Betaproteobacteria than either is to Deltaproteobacteria (to the point where the Beta-, Gamma-, and Alphaproteobacteria are now classified as phylum Pseudomonadota, with Deltaproteobacteria making up their own phylum Myxococcota). Chlorobi are not closely related to any of the Proteobacteria that their Cyc2 sequences group with. Epsilonproteobacteria (recently renamed Campylobacterota) sequences are nested within the zetaproteobacterial sequence diversity, suggesting that the common ancestor of epsilonproteobacterial Cyc2 was transferred into Epsilonproteobacteria from Zetaproteobacteria. The relationships recovered on the species tree are in good agreement with prior studies on the bacterial species tree (e.g., see references 16 and 17). For Epsilonproteobacteria whose placement has been uncertain, we recover a placement in agreement with reference 17, basal to the other proteobacterial groups in our tree.

Even within groups of sequences representing one clade of organisms, signatures of horizontal gene transfer in the Cyc2 tree are readily observable. In agreement with prior work (see their Fig. 1 and 2 in reference 9), we observe that the tree of Cyc2 sequences in photoferrotrophic Chlorobi does not reflect species tree relationships: genus *Chlorobium* is monophyletic on the 16S tree, but the Cyc2 sequences in the *Pelodictyon luteolum* group within the *Chlorobium* Cyc2 diversity (for the 16S and Cyc2 trees with no collapsed nodes, see Fig. S1 and S4, respectively). In particular, the Cyc2 sequences in *C. phaeovibrioides* and *P. luteolum* are closely related, while on the 16S tree *C. phaeovibrioides* groups with other members of genus *Chlorobium* to the exclusion of *Pelodictyon*. The relationships between Cyc2 orthologs in Chlorobi are recovered with high support values, suggesting that they are not an artifact of phylogenetic uncertainty due to the relatively small amount of phylogenetic data (in Cyc2, a single protein on average about 400 amino acids in length). Therefore, photoferrotrophs are polyphyletic within Chlorobi not only due to differential loss but also due to a horizontal gene transfer component.

**Fig 2:**
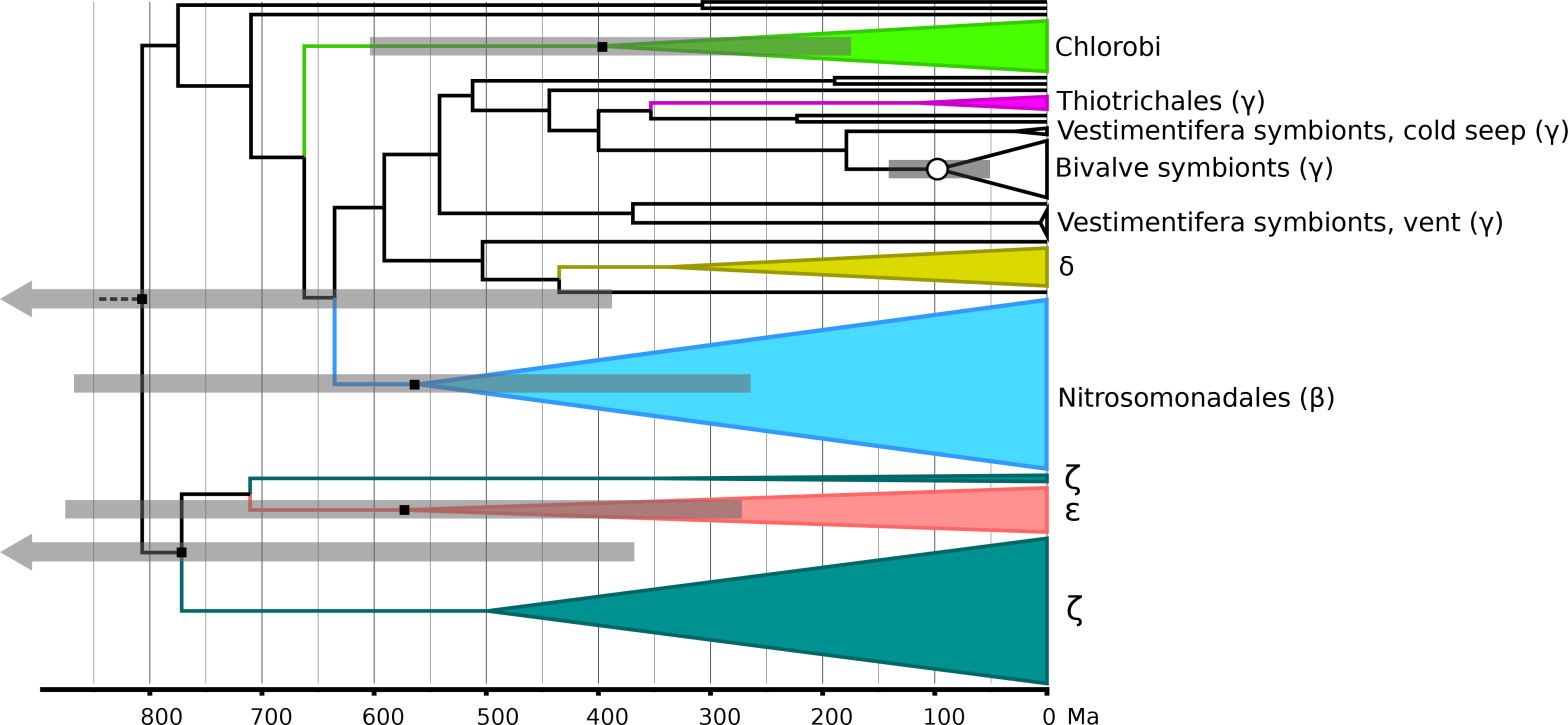
Bayesian relaxed molecular clock based on Cyc2 protein sequences. This representative instance shows mean ages under a uniform tree process prior and an uncorrelated clock model with rates on each branch drawn from a gamma distribution. The white circle marks the calibrated internal node, and black squares mark other key nodes discussed in this study. Gray bars represent the standard deviation of the posterior age distribution for key nodes (arrows signifying older bounds that lie off the edge of the figure), with 95% credible intervals shown in Table 1 to reflect the full range of likely dates. Greek letters refer to taxa falling in the following groups: β, Betaproteobacteria; γ, Gammaproteobacteria; ζ, Zetaproteobacteria (all three now classes in phylum Pseudomonadota); δ, Deltaproteobacteria (now phylum Myxococcota); ε, Epsilonproteobacteria (now phylum Campylobacterota). See Fig. S5 for the full chronogram (no groups collapsed), and see supplemental data at https://doi.org/10.5061/dryad.905qfttvf for the annotated tree file.

The general pattern of non-vertical relationships in the Cyc2 phylogeny suggests a high contribution of horizontal transfer to the overall tree structure. It is also worth noting that some parts of the tree suggest clustering according to environment, for example, the sequences from the Epsilonproteobacteria group within those from Zetaproteobacteria, and both groups occur in hydrothermal vent environments. Thus, one possible interpretation of the tree is that we are seeing a history of horizontal gene transfers between organisms inhabiting similar reduced aquatic environments, in addition to periods of vertical inheritance.

### Considerations for dating the Cyc2 tree

When it comes to the interval of time represented by the Cyc2 tree, the first-order observation that groups on the tree represent only shallow clade structure suggests that the interval of time covered by them is relatively short in geological terms. Sequences belonging to major groups cover only a small subset of their diversity, usually dominated by one or two smaller subclades (e.g., *Chlorobium* within Chlorobi and Gallionellaceae within Nitrosomonadales) where lower-level taxonomic assignment is available. While dating these shallow clades is difficult in the absence of a diagnostic paleontological or geochemical record, they are clearly younger than the major bacterial groups they belong to. As they still cover considerable tree depth (see Fig. 1a; e.g., about half between the tips and the root for the group sequences belonging to Gallionellaceae), it similarly suggests a relatively young age for the common ancestor of extant Cyc2 diversity. In contrast, the corresponding clades on the species tree are separated by long branches and make up little tree depth (see Fig. 1b).

Regarding photoferrotrophs in particular, the tree structure clearly rejects the suggestion of any direct relationship between modern photoferrotrophy in phylum Chlorobi and photoferrotrophs in their purported Archean golden age. Imagine what the tree would look like if the diversity of Cyc2 in extant photoferrotrophic Chlorobi extended all the way to the Archean. In that extreme case, the whole diversity of microaerophilic iron oxidizers such as Gallionellaceae and Zetaproteobacteria should nest within photoferrotrophic Chlorobi on the Cyc2 tree, as their aerobic metabolisms would only be expected to arise starting in the Proterozoic after increased oxygenation of the atmosphere. That is plainly not the tree structure we observe, with Chlorobi representing only a shallow group on the Cyc2 tree and with all other diversity falling outside of it. This is not a feature of misrooting: see Materials and Methods for how the root has been determined, but note also that the shallowness of the group alone makes it very unlikely that the tree should be rooted within this group.

A relaxed Bayesian molecular clock analysis allows for more quantitative testing of these heuristic arguments. Estimating divergence times in the tree requires meaningful prior choices in addition to the sequence data, and these prior choices can be difficult to make in microbial trees with rampant horizontal transfer: tree priors modeling divergence events as consecutive speciation events (such as the birth-death prior) are not necessarily appropriate since many branches traverse multiple lineages through transfer, and calibrations on internal nodes are hard to come by due to a lack of diagnostic fossil or geochemical record in most groups of microbes. Choices of tree prior (the prior on divergence dates of internal nodes) and root prior (the prior on the age of the root) have been discussed above (see Materials and Methods), but a more detailed overview of the one applied internal node calibration is appropriate here, since it is itself part of the phylogenetic results.

In addition to iron oxidizers considered in previous studies, we found orthologs of Cyc2 in gammaproteobacterial endosymbionts of lucinid bivalves (Lucinidae) whose radiation is constrained in time by the availability of suitable seagrass meadows starting in the Cretaceous, providing the reduced environment where lucinids’ characteristic symbiosis with chemotrophic symbionts that oxidize the reduced species in the sediment would have been instrumental (27). Thus, we were able to use the well-dated record of lucinid bivalves (28) to time-calibrate the relevant node in Cyc2 phylogeny. Cyc2 sequences in lucinid endosymbionts group together in the tree, and previous studies have suggested that the corresponding bacterial group forms a monophyletic clade (Fig. 2 in reference 29). The most complete phylogenetic and ecological study of this group published (30)—proposing the group as “Ca. Thiodiazotropha”—only observed its members in symbioses with lucinid bivalves. While endosymbionts are acquired from the environment in lucinid bivalves and there is no faithful vertical co-evolution between the host and the symbiont at the species level (24), this evidence suggests that members of the group specifically enter into symbioses with lucinid bivalves and thus that their last common ancestor was also a lucinid endosymbiont. That allows us to impose a lower bound on the node representing this last common ancestor: it should not predate the appearance of lucinid bivalves. In a previously published molecular clock of Lucinidae well constrained by fossil calibrations, the divergence of Lucinidae from its sister group takes place 170 million years ago (28). Given that the establishment of the symbiotic relationship could in principle have taken place in stem lucinids, we use this total group age for Lucinidae as a conservative older bound for the age of the last common ancestor of lucinid endosymbiont Cyc2 sequences.

### Molecular clock outcomes and sensitivity testing

**Fig 3:**
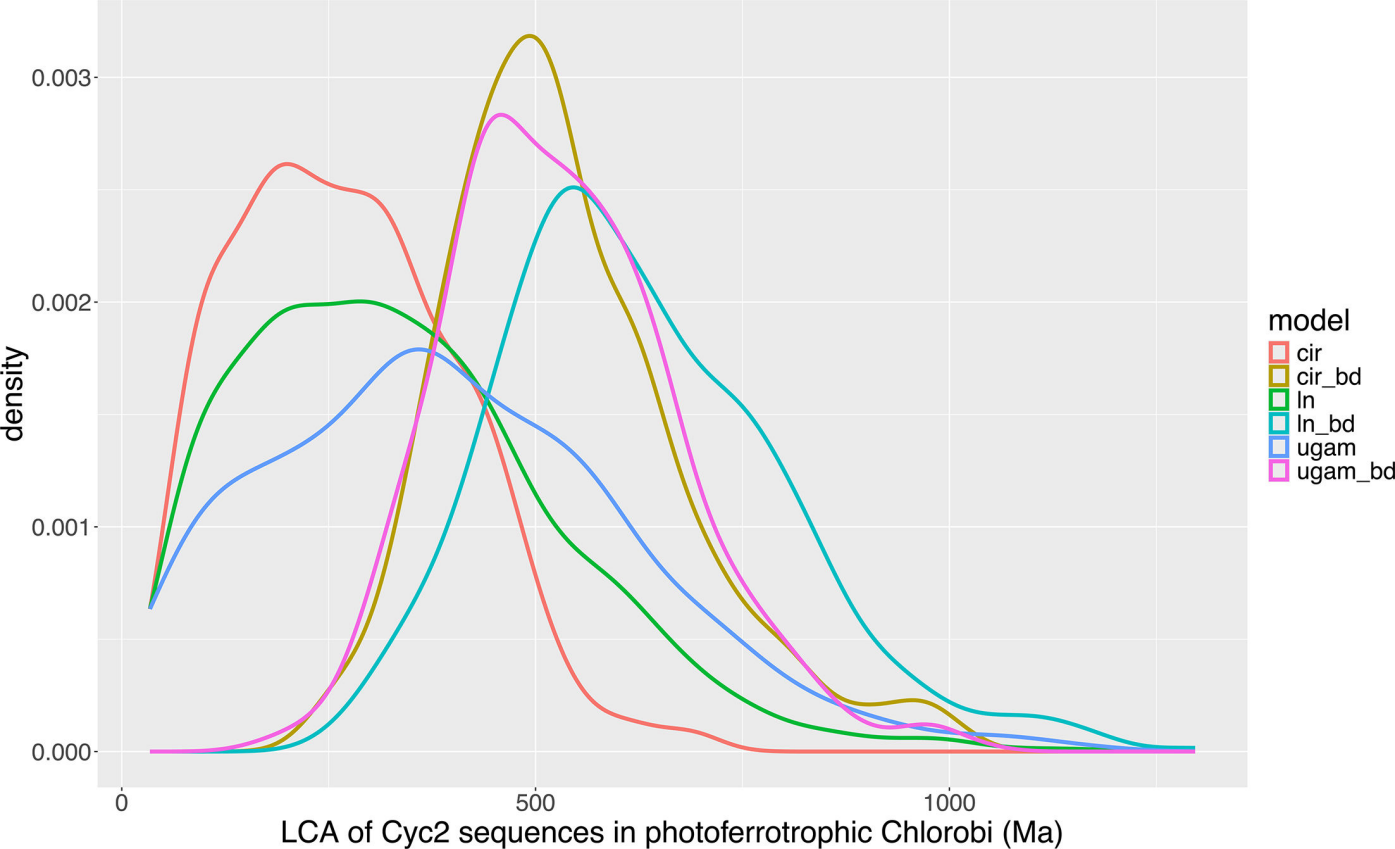
Overlaid posterior probability distributions for the age of the last common ancestor of Cyc2 sequences in photoferrotrophic Chlorobi under all combinations of clock models and tree prior processes, summarizing the full range of dating uncertainty. Clock models: cir, autocorrelated clock with branch rates based on the CIR process; ln, autocorrelated clock with branch rates drawn from a log-normal distribution; ugam, uncorrelated clock with branch rates drawn from a gamma distribution. Clocks with “bd” have been run under a birth-death prior, while those without that marker use a uniform tree process prior.

In runs under the posterior (see Table 1 for a summary of key node ages and Fig. 2 for a representative clock), the most general pattern is that clocks using a birth-death prior rather than a uniform prior on divergence dates produce significantly older ages for internal nodes. This is likely because the node constrained by the internal calibration appears much shallower in the tree (i.e., at lower relative tree depth) under the birth-death prior, and the full depth of the tree is therefore estimated to be greater in absolute terms. For example, the mean age of the last common ancestor of Cyc2 sequences in photoferrotrophic Chlorobi falls between 270 and 400 Ma for runs with the uniform prior, but between 530 and 625 Ma for runs with the birth-death prior. However, for the purposes of testing the hypothesis of early origin for this group, it is crucial to observe that under no model does a significant fraction of the probability mass exceed 1 Ga: see Fig. 3 for full posterior probability distributions for the age of this node under all models. In other words, even though the tree prior process considerably impacts ages of internal nodes (likely due to the relative lack of sequence data, as Cyc2 has about 400 amino acids in most organisms), its influence on younger bounds of node ages is more profound than on older bounds.

**TABLE 1:** A summary of age estimates for key nodes on the Cyc2 tree^*a*^

	Age estimate (Ma) for the last common ancestor of Cyc2 sequences in key groups on the tree, mean (95% credible interval)					
	ugam	ugam bd	ln	ln bd	CIR	CIR bd
Root	806 (1,764–118)	1,407 (2,134–806)	536 (1,256–96)	1,323 (2,144–708)	386 (823–101)	1,152 (2,012–581)
Photoferrotrophic Chlorobi	397 (928–50)	532 (859–297)	338 (781–64)	625 (1,047–334)	270 (552–66)	534 (963–298)
Nitrosomonadales (incl. Gallionellaceae)	570 (1,265–85)	828 (1,281–471)	399 (912–72)	767 (1,241–420)	309 (635–80)	668 (1,085–394)
Epsilonproteobacteria (Campylobacterota)	573 (1,287–85)	941 (1,495–534)	405 (943–76)	882 (1,416–486)	317 (698–84)	806 (1,369–400)
Zetaproteobacteria	771 (1,647–116)	1,332 (1,999–756)	512 (1,205–91)	1,257 (2,024–694)	374 (797–98)	1,089 (1,882–541)
Gammaproteobacterial lucinid endosymbionts	97 (168–14)	142 (169–81)	93 (166–19)	140 (169–82)	95 (165–26)	145 (170–89)

^*a*^ Note that these estimates are not for the last common ancestor of organisms in each group, but rather the last common ancestor of Cyc2 sequences present in members of the group. Clock models: “ugam” denotes an uncorrelated model with rates on each branch drawn from a gamma distribution; “ln” denotes an autocorrelated model with rates drawn from a log-normal distribution; and “cir” denotes an autocorrelated model with rates based on the CIR process. Addition of “bd” signifies that the clock was run under the birth-death tree process prior; otherwise, the uniform prior was used.

In general, greater uncertainties on younger bounds than older bounds are to be expected, as the one internal node calibration used only constrains its older bound: the substitution rate is constrained to be above a certain value and does not allow branches to stretch arbitrarily far into the past. This offers a one-sided test for the hypothesis of ancient origin for the iron oxidation process in question. Note also that the presence or absence of correlation between substitution rates on adjacent branches has less of an impact on divergence dates than the prior process. That observation is particularly important since we have imposed a calibration on a clade of endosymbionts, where substitution rates could substantially differ from those in related free-living lineages. Autocorrelated and uncorrelated clocks have a different capacity to take such a transition into account, and it should increase overall confidence in these dating estimates that outcomes of autocorrelated and uncorrelated clocks do not contrast greatly with each other.

Other clades of modern iron oxidizers on the tree show patterns similar to photoferrotrophic Chlorobi. Mean ages of 660–830 Ma are calculated for the last common ancestor of Gallionellaceae sequences under models with a birth-death prior, and 300–570 Ma under models with a uniform prior. For zetaproteobacterial Cyc2 sequences whose last common ancestor stretches deeper into the tree, the age estimates range between 370 and 780 Ma for the uniform prior and between 1,080 and 1,340 Ma for the birth-death prior. Full chronograms showing the 95% credible intervals for all internal nodes are shown in Fig. S5–S10.

## DISCUSSION

The relaxed molecular clock analysis shows that iron oxidation with Cyc2 in modern photoferrotrophic Chlorobi is a relatively young trait, originating in the late Neoproterozoic or the Phanerozoic. This result also agrees with previous estimates of relatively recent origin for the clade of Chlorobi in which photoferrotrophy is present. It is inconsistent with views of the current process of photoferrotrophy as a direct descendant of photoferrotrophic iron oxidation in the Archean and Paleoproterozoic that may have played a role in the deposition of extensive BIFs during these times.

Furthermore, timing estimates for the last common ancestor of Cyc2 sequences in other major microbial iron oxidizers such as Gallionellaceae and Zetaproteobacteria are also relatively recent across all clock runs, with the earliest likely emergence of Cyc2-based iron oxidation in Neoproterozoic in Gallionellaceae and in Mesoproterozoic in Zetaproteobacteria. Even though fossils morphologically similar to modern chemolithotrophic iron oxidizers date back to at least the Paleoproterozoic (e.g., see references 31 and 32), the recent origin of the modern clades suggests that these early fossils do not represent known extant groups. While chemolithotrophic iron oxidation and photoferrotrophy as metabolisms are undoubtedly very ancient, the taxonomy of responsible organisms has changed profoundly through time.

In our effort to date the history of Cyc2, we have chosen to be conservative in the selection of priors and inclusive in testing the impact of a broad variety of molecular clock models on the divergence date estimates. This is to recognize that different clocks often yield very different ages, and we do not have a good way to argue for the use of one set of parameters over another for the very reason why ages in microbial natural history are hard to estimate: rock record calibrations are scant, and our understanding of the underlying evolutionary process (impact of transfer vs vertical inheritance, estimate of substitution rates across branches in the distant past) is constrained. As we are dating events in the history of a single gene, the available sequence information is necessarily limited. Dating outcomes also depend on the chosen tree process prior: the birth-death prior models nodes as consecutive speciation events (though branches in this tree commonly traverse distantly related lineages owing to transfer), while the uniform prior assumes no knowledge of evolutionary process whatsoever and is sometimes dismissed because there is no known evolutionary process that would yield such a distribution of branching events (33). We have no way to decide between them, and thus, we have to live with considerable dating uncertainties. Nevertheless, sensitivity testing shows that even in the presence of these uncertainties and limited phylogenetic information, we can reject the hypothesis of taxonomic and ecological continuity across the long history of microbial iron oxidation on Earth. Rather, our molecular clock results show a recent origin of iron oxidation in extant microbial groups, as also reflected by the shallow clade structure of the tree alone.

Ecologically, the shallow clade depths could reflect the environmental history and bioavailability of iron: in a largely oxygenated world, niches with sufficient reduced iron are geographically restricted and ecologically fragmented, and they do not persist over geologically long periods of time. After a while, this electron donor becomes scarce, and organisms either adapt by loss of the pathway or they simply leave no descendants. However, there are always refugia somewhere in the ocean with sufficient reduced iron, even if an individual instance of that environment does not last particularly long. When reduced iron becomes locally abundant, Cyc2 (and potentially other machinery for iron oxidation) is shared by horizontal transfer by the bacterial populations in this environment, and it is broadly retained due to the useful metabolic trait it imparts. It is possible that retention of iron oxidation machinery would have been more consistent and patterns of vertical inheritance more pronounced if we examined their phylogeny in the Archean, when selection for being able to use reduced iron as an electron donor would have been more consistent in an ocean with plenty of reduced iron.

If the ecology of iron metabolism in the recent oceans is defined by its spotty availability, this could also be reflected in the evolutionary history and phylogenetic distribution of other traits partaking in iron metabolism. While Cyc2-dependent iron oxidation is useful where reduced iron is available, siderophores are commonly used for scavenging iron where it is unavailable (34). If the presence of this reverse condition is also somewhat variable in space and time, then loss, transfer, and re-acquisition of siderophore biosynthesis pathways could show similar evolutionary patterns. Indeed, for example, in Cyanobacteria, siderophore biosynthesis pathways show a patchy distribution: the production of a given siderophore is often polyphyletic, and even closely related taxa can produce different siderophores (35). Though re-constructing phylogenetic histories for particular siderophores is challenging owing to the promiscuity of the biosynthesis enzymes (35), these observations are definitely consistent with frequent departures from vertical inheritance. Shallow clade depth and frequent radiation, loss, and transfer may characterize a general pattern in the evolution of iron metabolism machinery.

Regarding the ecological dynamics influencing the evolutionary history of iron utilization, it is worth adding that many of the organisms appearing in this study of Cyc2 also seem to oxidize sulfur. For example, the gammaproteobacterial endosymbionts within lucinid bivalves that were essential for dating this history have been previously identified as carrying out sulfur oxidation for their hosts. Though this is not an issue for dating purposes as our approach is agnostic of the function of Cyc2 in these microbes, it has not been experimentally demonstrated to carry out iron oxidation in them. Beyond the gammaproteobacterial endosymbionts of lucinid bivalves, the green sulfur bacteria (Chlorobi) on the tree are so named because they most commonly rely on sulfide as an electron donor for photosynthesis, and the Epsilonproteobacteria are largely sulfur-oxidizing chemotrophs. In future work, it may be illuminating to consider further the reasons behind the taxonomic overlap between metabolisms utilizing reduced iron and sulfur: is it simply an ecological reflection of organisms living in environments with reduced fluids where each species is present, or is the reason a more profound overlap between the adaptations and machinery necessary for these metabolisms? These questions will become simpler to answer once we understand more clearly how the evolutionary histories of iron and sulfur metabolisms are separate or intertwined.

**Fig 4:**
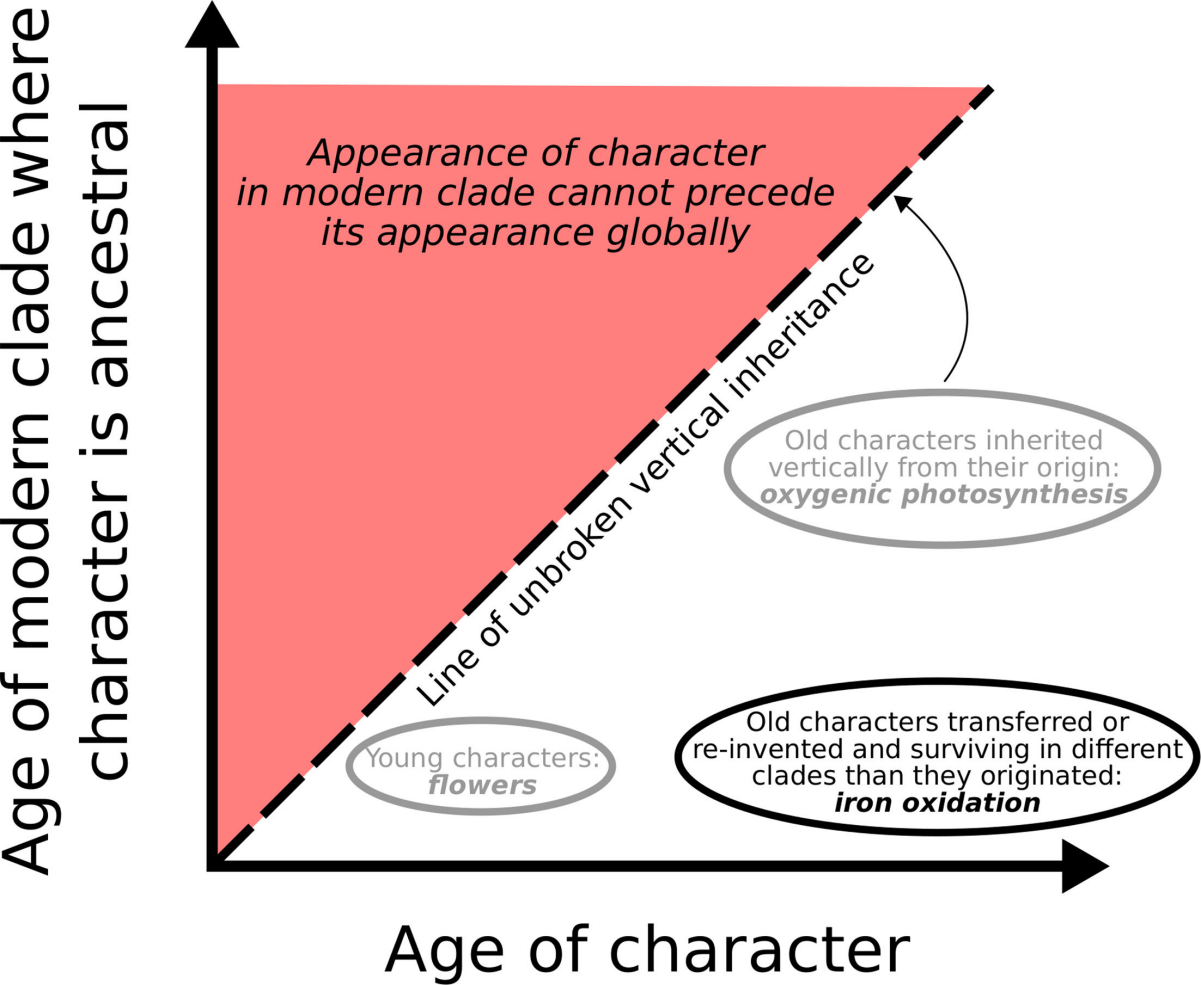
Schematic depicting examples of traits with varying levels of clade fidelity. The clade fidelity of a character is defined by its vertical distance from the 1:1 line (dashes) compared to its distance from the *x*-axis. Characters with low clade fidelity (such as microbial iron oxidation) plot close to the *x*-axis, as modern clades with the character are much younger than the character itself; characters with high clade fidelity (such as oxygenic photosynthesis) plot closer to the 1:1 line or even onto it. Note that it makes sense to talk about the clade fidelity of a character mainly over long timescales where radiation, extinction, and transfer are likely to occur. In the microbial world (such as for iron oxidation), geological evidence of a trait’s presence is often necessary to infer its age, as evolutionary inferences made using the genetic record in extant organisms do not give access to extinct lineages.

In describing the evolutionary and phylogenetic history of these metabolisms, we have proposed defining a trait’s “clade fidelity” (36): its tendency toward vertical inheritance and a stable taxonomic distribution over time, as opposed to frequent transfer and change in its taxonomic distribution. Microbial iron oxidation considered here is a clear example of a trait with low clade fidelity: throughout its history, it is frequently transferred between clades or lost and regained. Some clades with the trait likely become extinct, while new clades become associated with it, and at any given time, the trait’s history in contemporary clades is shallow. For comparison (Fig. 4), this is in striking contrast to a trait like oxygenic photosynthesis, which evolved once in an ancestor of modern cyanobacteria and is exclusively present in the descendants of this organism, having been faithfully vertically inherited for well over two billion years (37).

On a planetary scale, there may be some selection for low clade fidelity on traits whose usefulness is episodic. A type of iron oxidation we considered in particular detail is photoferrotrophy, and it is interesting to note that the finding of its relative youth as carried out in the modern by some green sulfur bacteria parallels the relatively shallow histories of other forms of anoxygenic photosynthesis (for a summary, see reference 37). Unlike oxygenic phototrophs, anoxygenic phototrophs are limited by the variable abundance of the electron donor. It is striking, therefore, that forms of anoxygenic photosynthesis would show low clade fidelity, whereas oxygenic photosynthesis with its more reliable substrate exhibits the highest possible clade fidelity, having only evolved once and having apparently never been transferred to another microbial lineage. This is not to say that the reliable abundance of substrate causes high clade fidelity, but rather that a metabolism with high clade fidelity might not be able to survive unless consistently favored over geological time, including by the abundance of substrate. There may have been many forms of photoferrotrophy in the past that, for whatever reason (complex underlying machinery, lack of modularity or potential to be integrated with other metabolisms), showed high clade fidelity, but these forms would be more likely to go extinct, given their limited resilience to contexts where photoferrotrophy was not favored. A form of photoferrotrophy with low clade fidelity, distributed in a set of organisms and localities, is more likely to survive and subsequently radiate again when favorable conditions emerge.

Such a model assumes that selection operates not only on organisms themselves but also on biotic processes. This view has recently been popularized by the “It’s the song not the singer” theory initially proposed by Doolittle and Booth in the context of host-symbiont systems (38) but subsequently generalized to broader interactions between organisms in the ecosystem and even biogeochemical cycles (39). This theory emphasizes that interactions between organisms or processes mediated by them (“songs”) often outlast individual clades (“singers”) and should be considered proper units of selection themselves. If that is the case, it is easy to imagine clade fidelity as one of the main aspects of a trait or process to be under selection. Processes with high clade fidelity are contingent on the survival of individual clades in which they occur, and both are more likely to suffer from extinction and less likely to re-emerge convergently thereafter.

From a planetary perspective, the clade fidelity of a trait could even have implications for the robustness of the biosphere in which that trait participates. This is easiest to imagine if the trait is a metabolic ability to carry out a biogeochemical process which participates in a web of biotic interactions. It thus influences other organisms as well as the collective they make up together: the biosphere as a whole, whose stability over Earth history may have included a small positive contribution from the low clade fidelity of microbial iron oxidation.

We have suggested the clade fidelity framework as a means of interpreting our observations on the evolution of microbial iron oxidation and some other microbial metabolisms discussed above. However, it also predicts that other genes with functional niches that are discontinuous in space and time would show evolutionary histories consistent with low clade fidelity. Further work will show if this view is indeed broadly applicable to the evolution of microbial metabolisms.

To summarize the history of microbial iron oxidation from the Archean to the present in Doolittle’s terms, the singers have changed, but the song has endured. It is uncertain whether Cyc2 itself played a role in microbial iron oxidation before the emergence of the groups currently using it: Cyc2 may have been carried in the more distant past by lineages that left no extant descendants, yet transferred their iron oxidation machinery into lineages that did. Alternatively, earlier iron oxidation machinery could have been unrelated. Either way, when considering microbial iron oxidation in the geological past, it should be assumed that it transcended its modern taxonomic distribution; for traits of low clade fidelity, looking to modern organisms as proxies of past process is particularly perilous.

## Data Availability

The supplemental data files for this article, including all alignment, tree, and chronogram files, can be accessed on Dryad at https://doi.org/10.5061/dryad.905qfttvf.

## SUPPLEMENTAL MATERIAL

Supplemental figures aem.01662-24-s0001.docx Figures S1 to S10.

ASM does not own the copyrights to Supplemental Material that may be linked to, or accessed through, an article. The authors have granted ASM a non-exclusive, world-wide license to publish the Supplemental Material files. Please contact the corresponding author directly for reuse.
