# Supplementary material for: Recent origin of iron oxidation in extant microbial groups and low clade fidelity of iron metabolisms
Source: Appl Environ Microbiol. 2025 Aug 12;91(9):e01662-24. doi: 10.1128/aem.01662-24 (PMC12442345; doi:10.1128/aem.01662-24)
Supplement: Supplemental figures — Figures S1 to S10. [file aem.01662-24-s0001.docx]

## Supplemental Figures


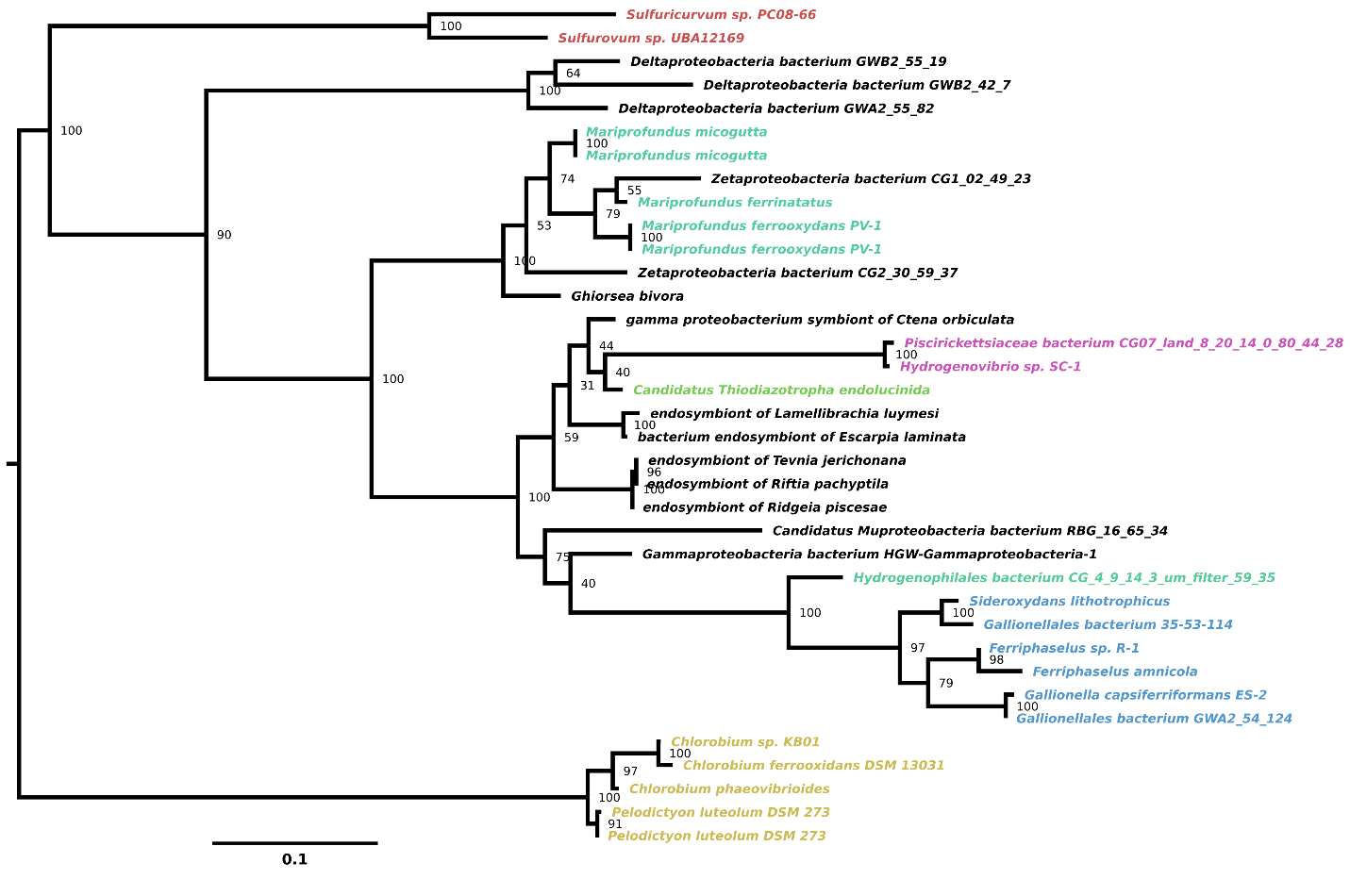


**Figure S1**. Species tree of organisms with Cyc2 built using the 16S small subunit ribosomal RNA sequences. Node labels represent ultrafast bootstrap support for the labeled bipartition. Taxa are colored according to taxonomic order (where assigned to an order).


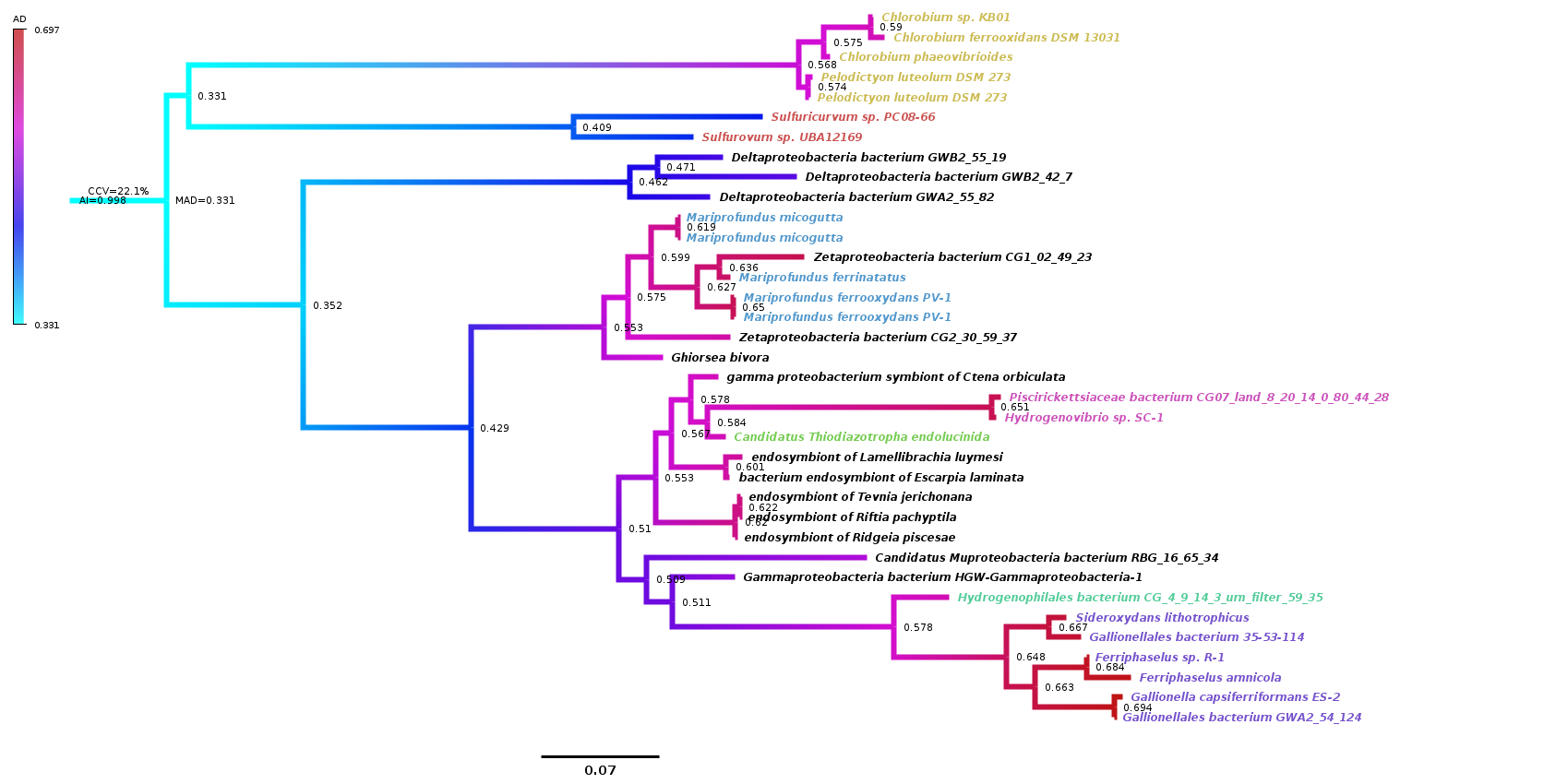


**Figure S2**. A visualization of rooting options for the 16S tree using minimum ancestor deviation (MAD), with the root placed to minimize the ancestor deviation (AD) value. Branches are colored according to AD values, and node labels represent AD values at the nodes. Taxa are colored according to taxonomic order (where assigned to an order).


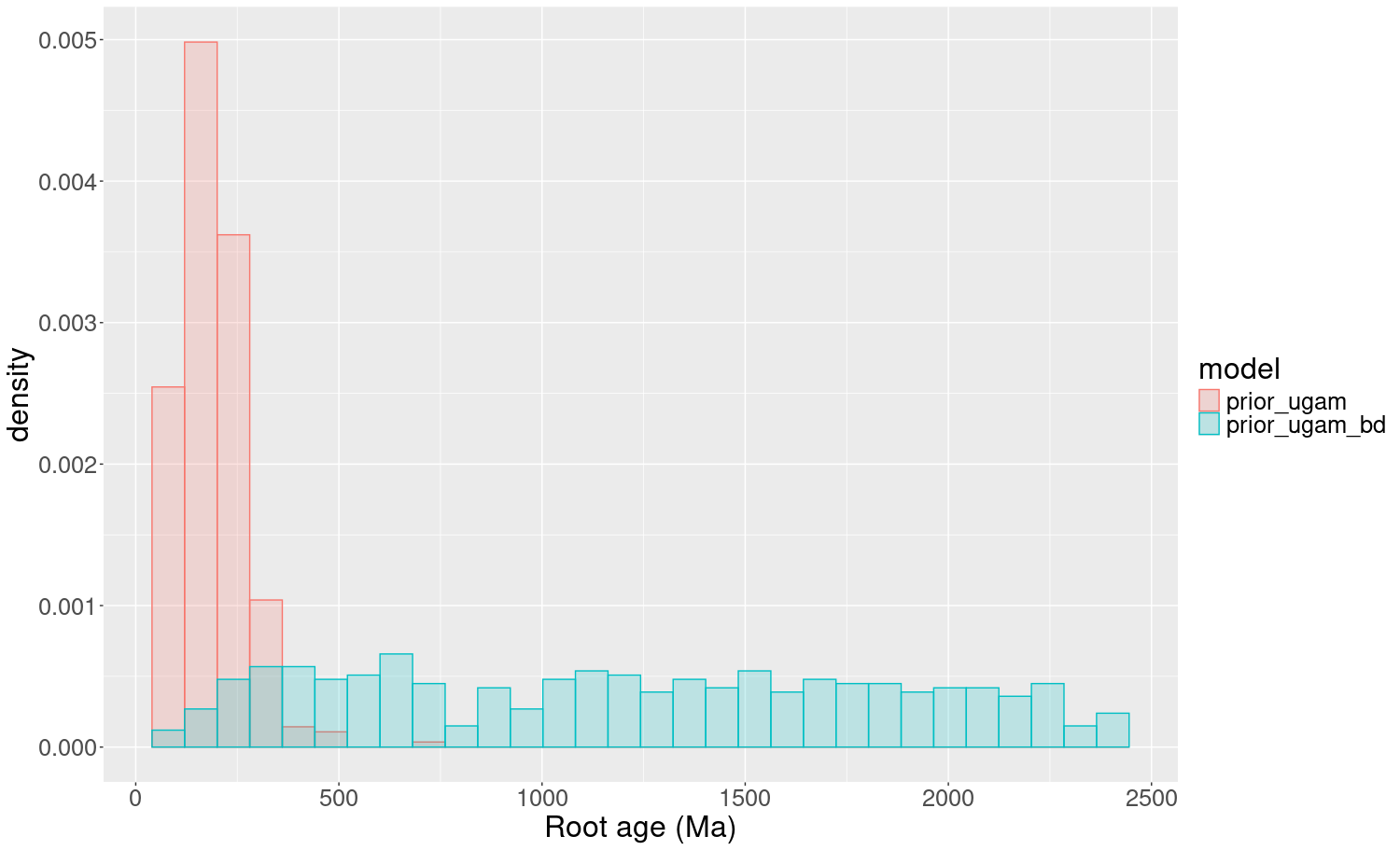
**Figure S3**. Root priors conditional on the internal node calibration and the tree process prior (uniform vs. birth-death prior on divergence dates).
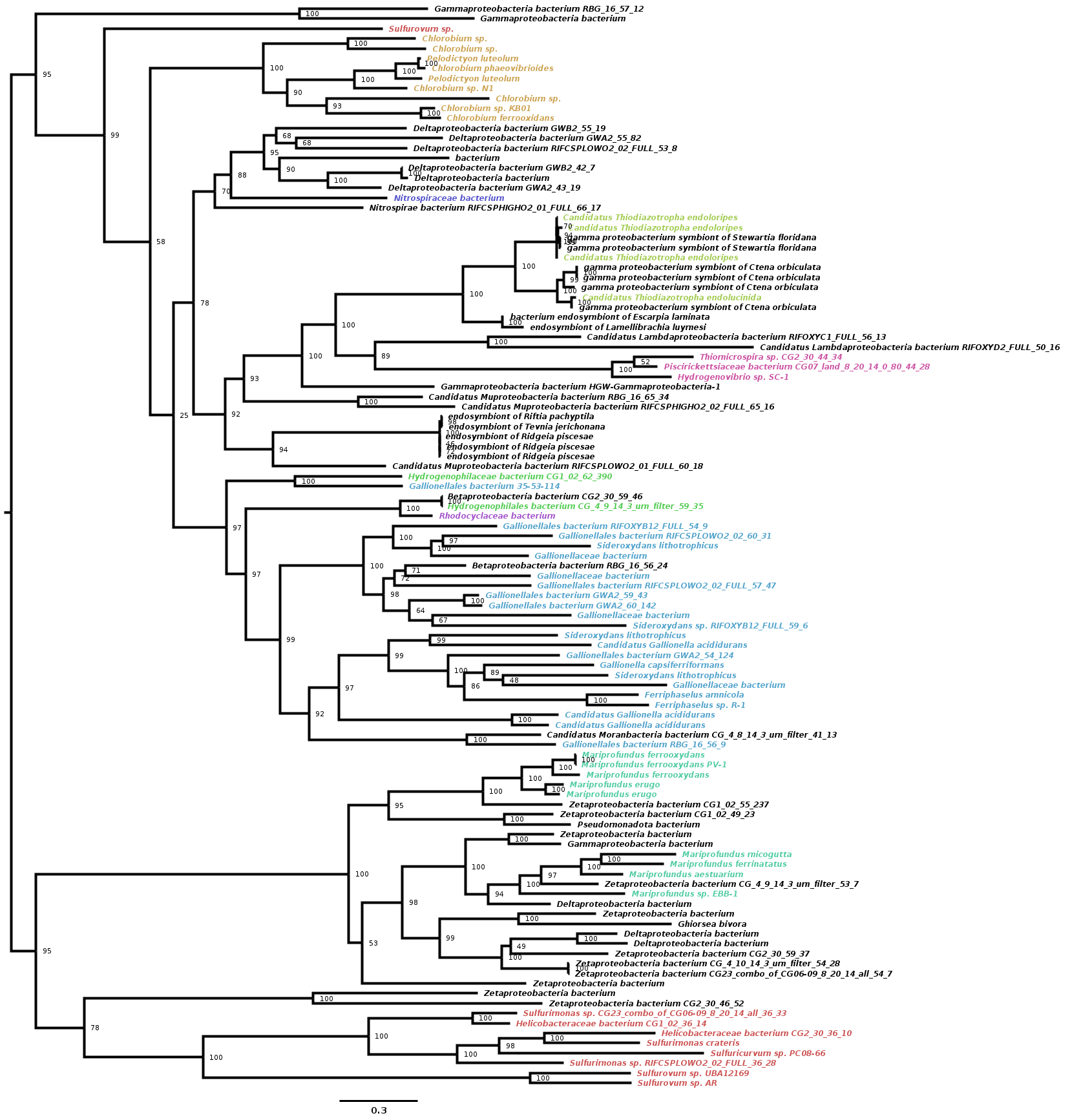


**Figure S4**. Full maximum-likelihood phylogenetic tree of Cyc2 sequences. Node labels represent ultrafast bootstrap support for the labeled bipartition. Taxa are colored according to taxonomic order (where assigned to an order).


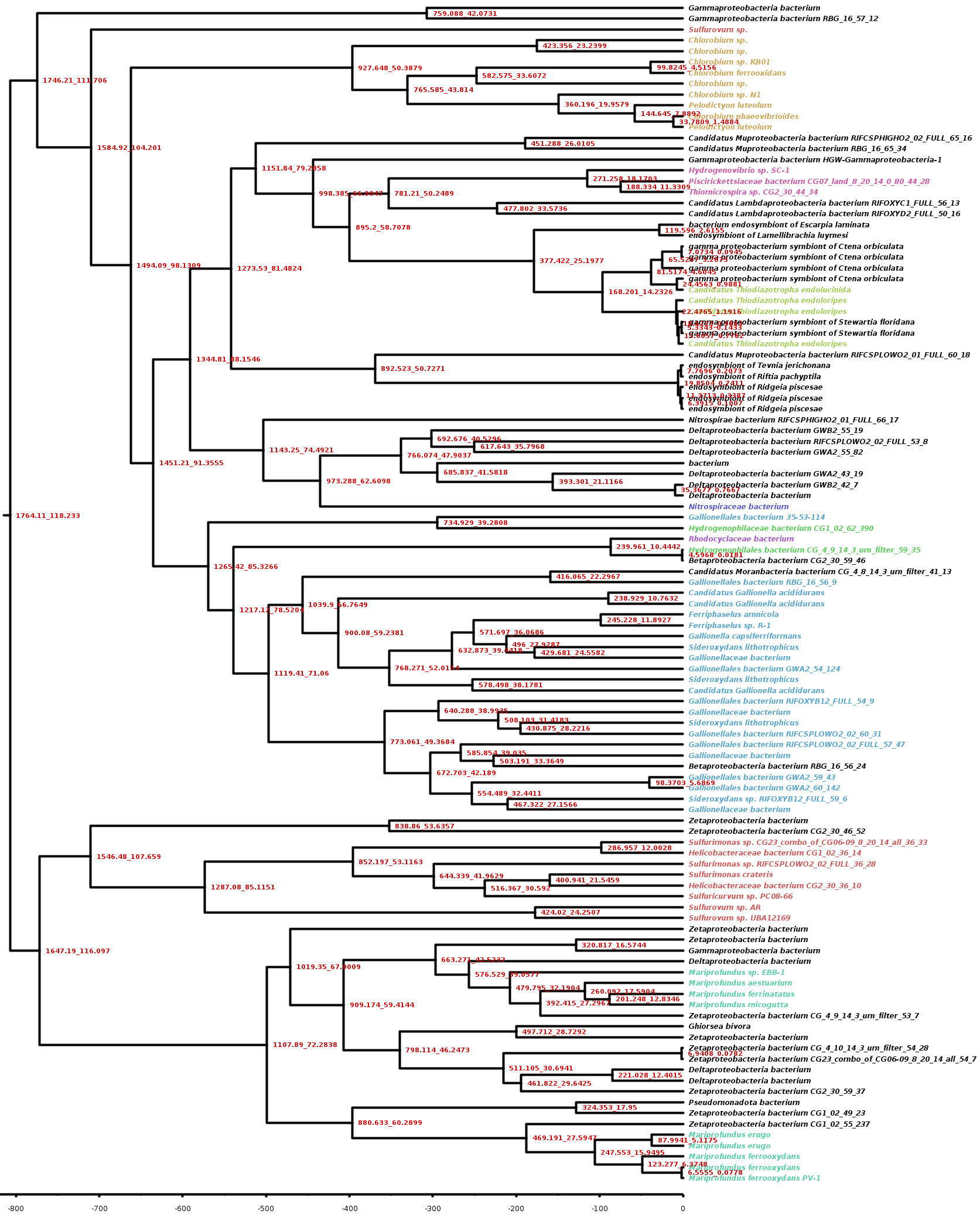


**Figure S5**. Full chronogram of Cyc2 sequences built using the uniform tree process prior and the uncorrelated gamma-distributed clock model. All ages in Ma. Node labels represent 95% posterior credible intervals for node age. Taxa are colored according to taxonomic order (where assigned to an order).


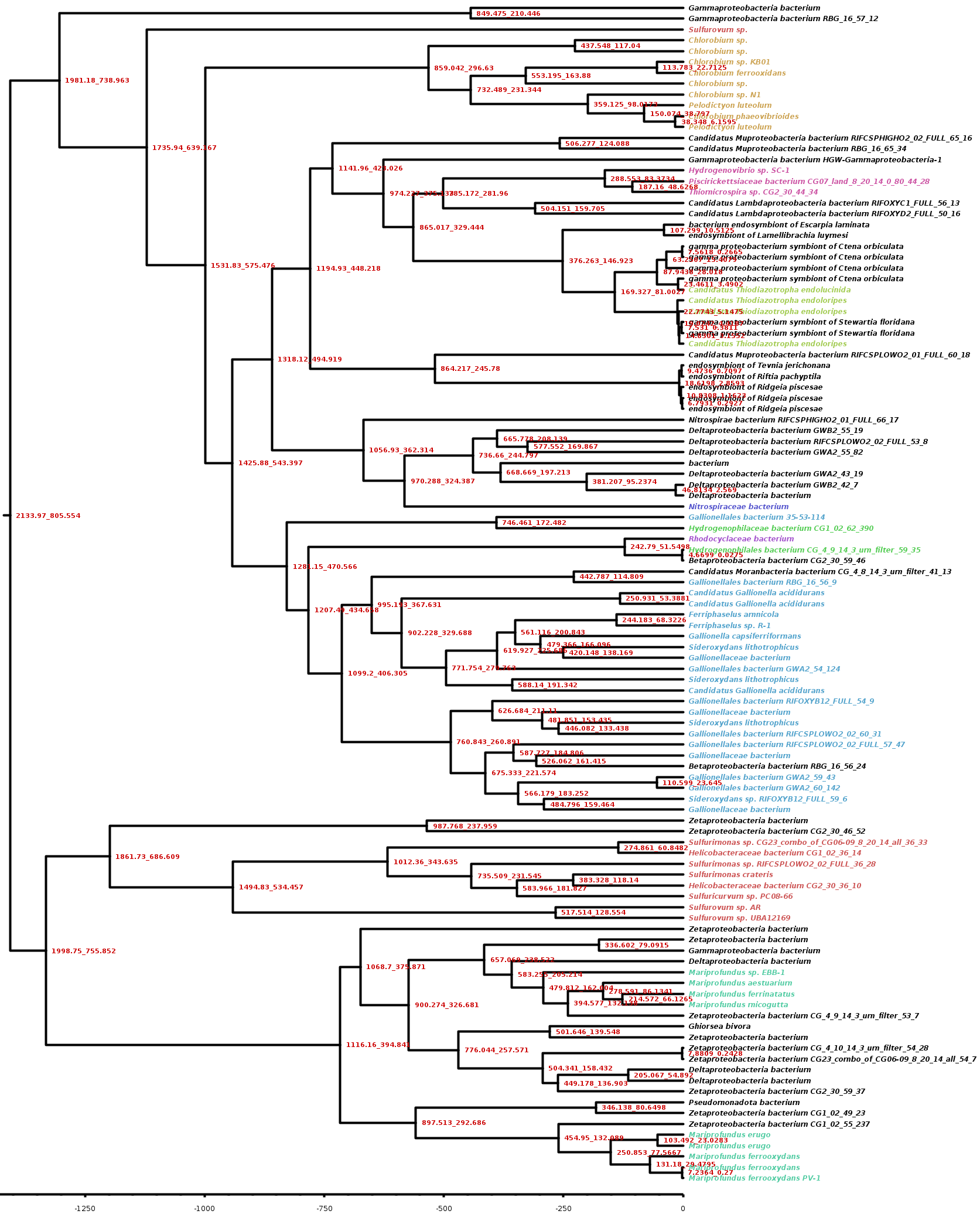


**Figure S6**. Full chronogram of Cyc2 sequences built using the birth-death tree process prior and the uncorrelated gamma-distributed clock model. All ages in Ma. Node labels represent 95% posterior credible intervals for node age. Taxa are colored according to taxonomic order (where assigned to an order).

**
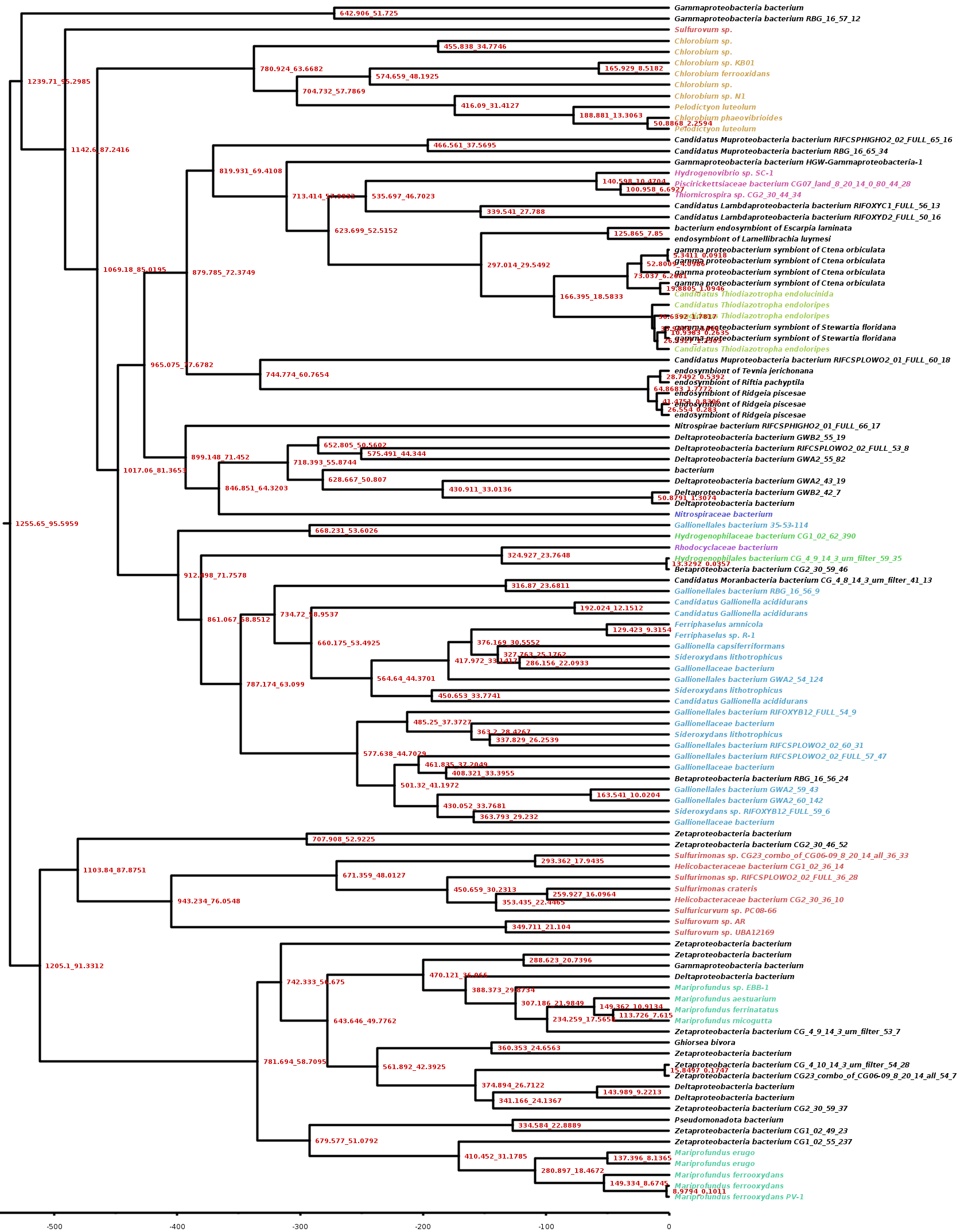
**

**Figure S7**. Full chronogram of Cyc2 sequences built using the uniform tree process prior and the log-normal clock model. All ages in Ma. Node labels represent 95% posterior credible intervals for node age. Taxa are colored according to taxonomic order (where assigned to an order).


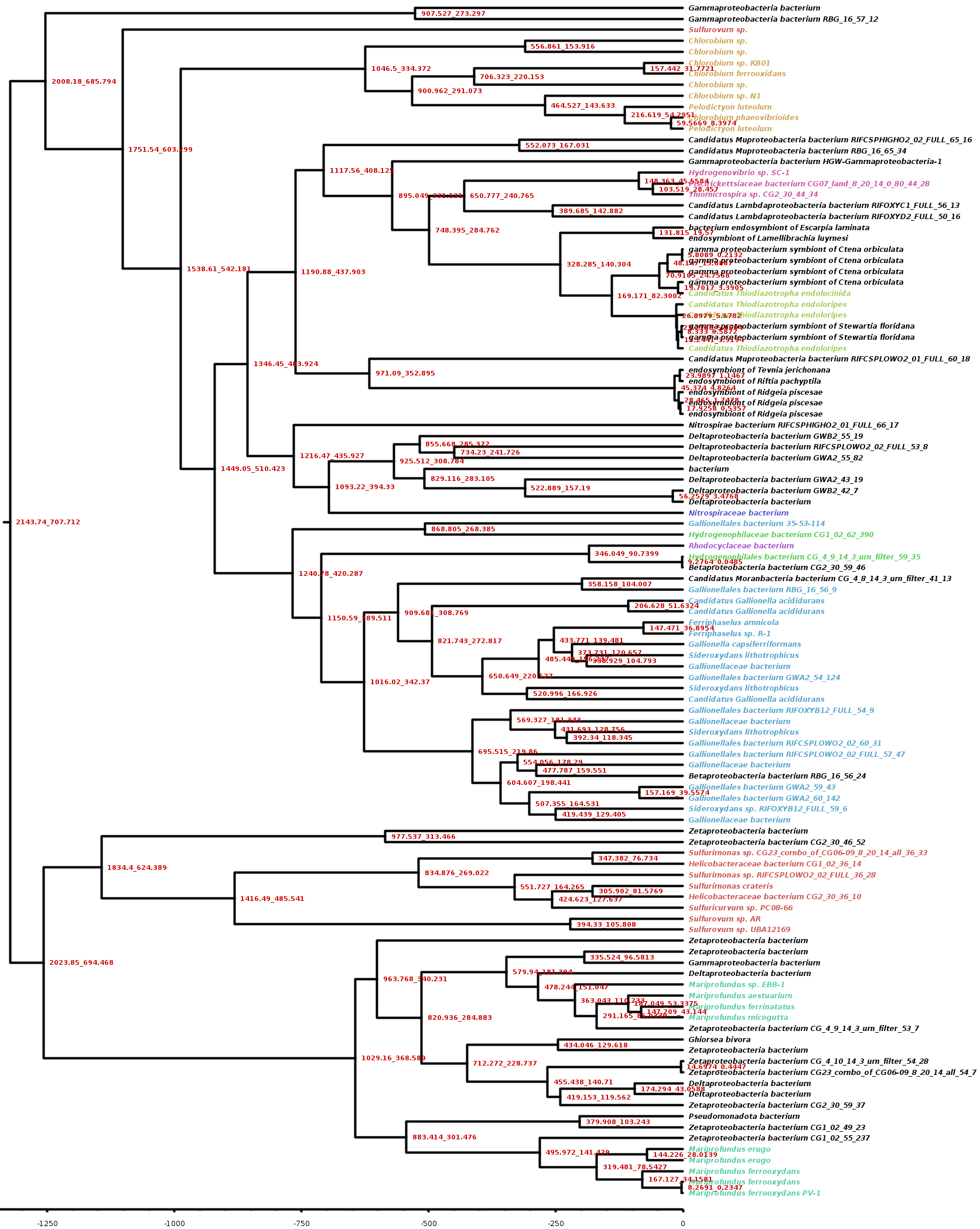


**Figure S8**. Full chronogram of Cyc2 sequences built using the birth-death tree process prior and the log-normal clock model. All ages in Ma. Node labels represent 95% posterior credible intervals for node age. Taxa are colored according to taxonomic order (where assigned to an order).


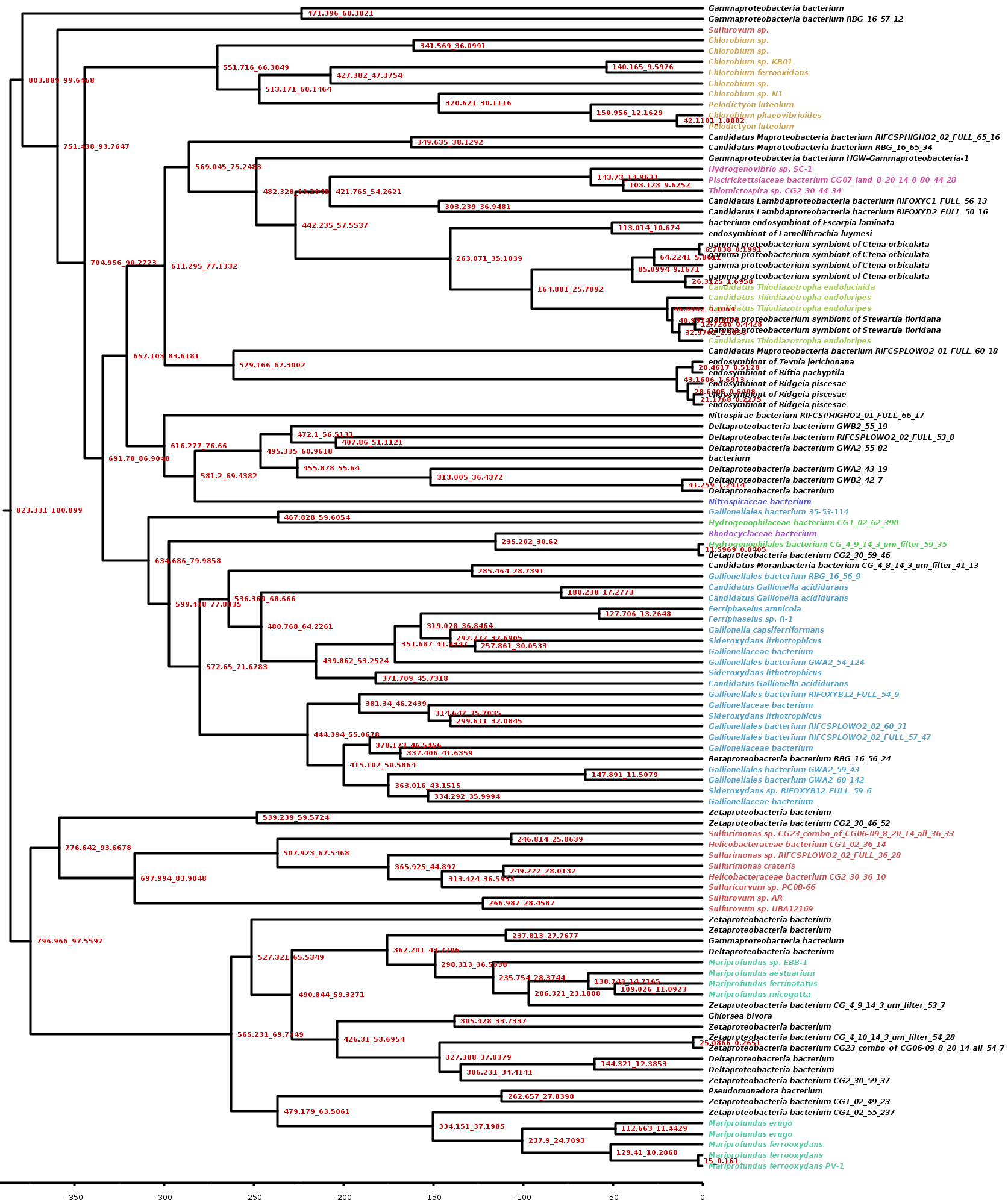


**Figure S9**. Full chronogram of Cyc2 sequences built using the uniform tree process prior and the CIR clock model. All ages in Ma. Node labels represent 95% posterior credible intervals for node age. Taxa are colored according to taxonomic order (where assigned to an order).


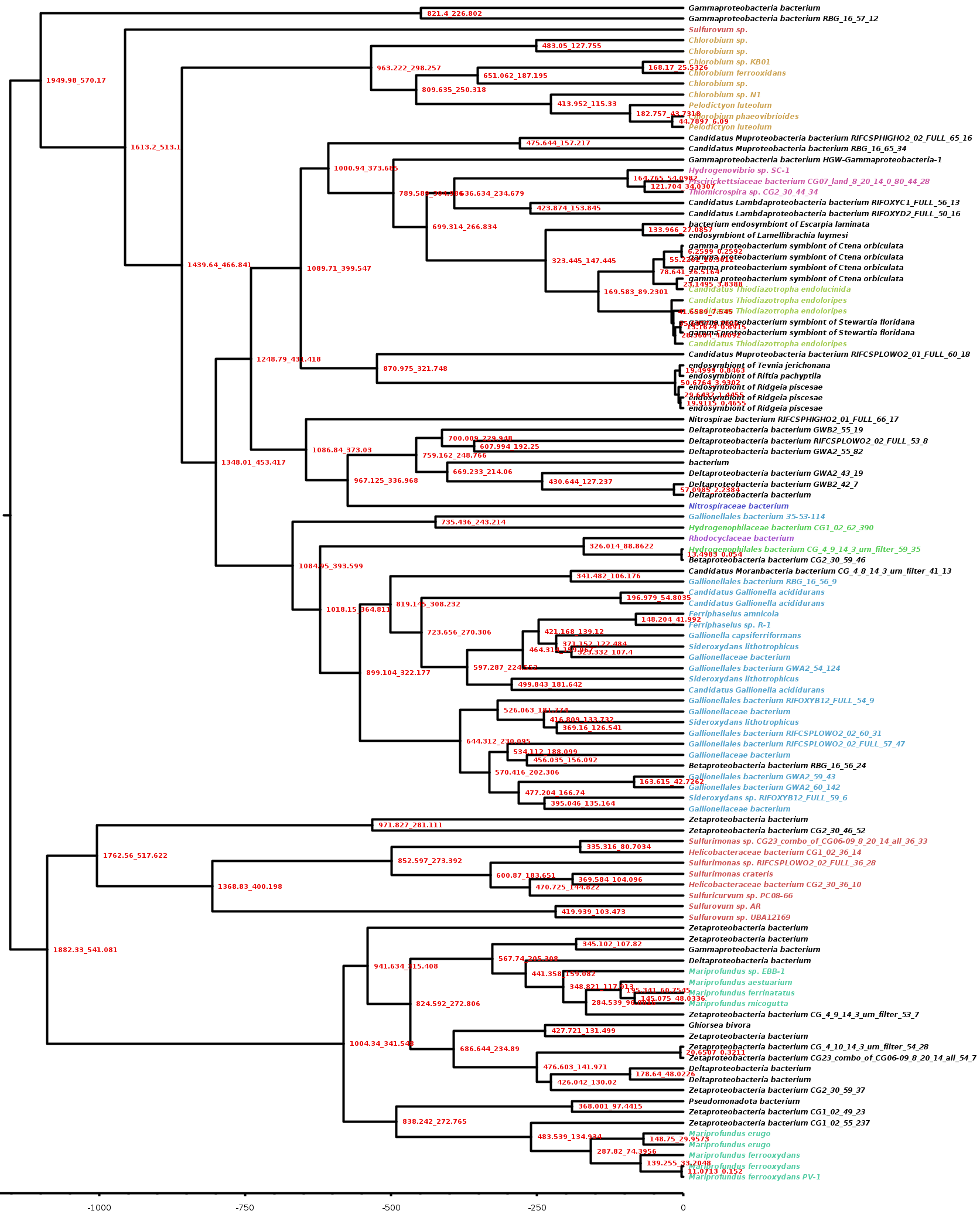


**Figure S10**. Full chronogram of Cyc2 sequences built using the birth-death tree process prior and the CIR clock model. All ages in Ma. Node labels represent 95% posterior credible intervals for node age. Taxa are colored according to taxonomic order (where assigned to an order).­­
